# Mesenchymal stem cells suppress neuronal apoptosis and decrease IL-10 release via the TLR2/NFκB pathway in rats with hypoxic-ischemic brain damage

**DOI:** 10.1186/s13041-015-0157-3

**Published:** 2015-10-17

**Authors:** Yan Gu, Yun Zhang, Yang Bi, Jingjing Liu, Bin Tan, Min Gong, Tingyu Li, Jie Chen

**Affiliations:** Children Nutrition Research Centre, Children’s Hospital of Chongqing Medical University, Chongqing, 400014 China; Ministry of Education Key Laboratory of Child Development and Disorders, Children’s Hospital of Chongqing Medical University, Chongqing, 400014 China; Chongqing Stem Cell Therapy Engineering Technical Centre, Children’s Hospital of Chongqing Medical University, Chongqing, 400014 China; Chongqing Key Laboratory of Translational Medical Research in Cognitive Development and Learning and Memory Disorders, Chongqing, 400014 China

**Keywords:** Mesenchymal stem cells, Hypoxic–ischemic brain damage, Oxygen and glucose deprivation, Toll-like receptor 2, Nuclear factor kappa B, Interleukin-1

## Abstract

**Background:**

Hypoxic–ischemic brain damage (HIBD) is a major cause of infant mortality and neurological disability in children. Many studies have demonstrated that mesenchymal stem cell (MSC) transplantation facilitates the restoration of the biological function of injured tissue following HIBD via immunomodulation. This study aimed to elucidate the mechanisms by which MSCs mediate immunomodulation via the key effectors Toll-like receptor 2 (TLR2) and interleukin-10 (IL-10).

**Results:**

We showed that TLR2 expression in the brain of HIBD rats was upregulated following HIBD and that MSC transplantation suppressed the expression of TLR2 and the release of IL-10, thereby alleviating the learning-memory deficits of HIBD rats. Following treatment with the specific TLR2 agonist Pam3CSK4 to activate TLR2, learning-memory function became further impaired, and the levels of nuclear factor kappa B (NFκB) and Bax expression and IL-10 release were significantly increased compared with those in HIBD rats that did not receive Pam3CSK4. *In vitro*, we found that MSC co-culture downregulated TLR2/NFκB signaling and repressed Bax expression and IL-10 secretion in oxygen and glucose deprivation (OGD)-injured adrenal pheochromocytoma (PC12) cells. Furthermore, NFκB and Bax expression and IL-10 release were enhanced following Pam3CSK4 treatment and were decreased following siTLR2 treatment in OGD-injured PC12 cells in the presence or absence of MSCs.

**Conclusions:**

Our data indicate that TLR2 is involved in HIBD and that MSCs decrease apoptosis and improve learning-memory function in HIBD rats by suppressing the TLR2/NFκB signaling pathway via a feedback mechanism that reduces IL-10 release. These findings strongly suggest that MSC transplantation improves HIBD via the inhibition of the TLR2/NFκB pathway.

## Background

Perinatal hypoxic-ischemic brain damage (HIBD) is a major cause of neonatal death that occurs in three to five out of every 1000 live births [1]. As many as 10 to 60 % of infants who experience HIBD die during the newborn period, and up to 25 % of the survivors of such injury exhibit a variety of serious neurological sequelae that decrease the quality of life of HIBD children and increase the economic and social burdens on their families [2, 3]. Because the pathogenesis of HIBD involves a series of complex processes [4, 5], it is unreasonable to expect any single intervention to provide a uniformly favorable outcome. Therefore, current therapeutic strategies typically focus on arresting the progression of one or more detrimental processes.

Based on developments from mesenchymal stem cell (MSC) studies, cell therapy has become a research focus for HIBD. Studies from several laboratories, including our own, have indicated that MSC treatment of ischemic brain injury provides beneficial effects [6–10]. In previous years, studies of MSC therapy for HIBD have focused on the multipotent differentiation properties of MSCs, with the aim of replacing the lost neurons and glial cells [11, 12]. However, administered MSCs cannot survive for long in the brains of recipients [13]; thus, increasing attention is being paid to their immunomodulatory functions in the injury microenvironment [14–16].

Toll-like receptors (TLRs) are transmembrane pattern recognition receptors (PRRs) [17] that form a family of innate immune system receptors that respond to pathogen-derived and tissue damage-related ligands [18]. TLR2 is a TLR family member that is known to play an important role in ischemic brain damage [19]. However, few studies have been performed to determine whether MSC transplantation regulates the TLR2 signaling pathway, thereby improving learning-memory function following HIBD.

The aims of the present study were to elucidate whether changes in the TLR2 expression level are associated with learning-memory function following HIBD and whether MSC transplantation regulates the TLR2 signaling pathway and promotes recovery from HIBD. First, we transplanted MSCs into HIBD rats and measured the changes in the TLR2 expression levels in the hippocampus. Next, we verified that TLR2 is an important factor in learning-memory function following HIBD using Pam3CSK4, which is a specific agonist of TLR2. Second, an oxygen-glucose deprivation (OGD) model was performed on adrenal pheochromocytoma (PC12) cells *in vitro* to simulate neuronal injury in HIBD rats *in vivo* to further confirm that TLR2 participates in MSC-mediated immunomodulation. Finally, we treated a co-culture system with Pam3CSK4 and siTLR2 to identify the mechanism underlying MSC-mediated neuroprotection via TLR2 signaling. Furthermore, we monitored the changes in the release of the cytokine interleukin (IL)-10 both *in vivo* and *in vitro.* This study might provide a new perspective revealing the immunomodulatory and neuroprotective properties of MSC therapy.

## Results

### MSC transplantation decreased the TLR2 expression, thus improving the learning-memory function of neonatal rats following HIBD

**Fig. 1:**
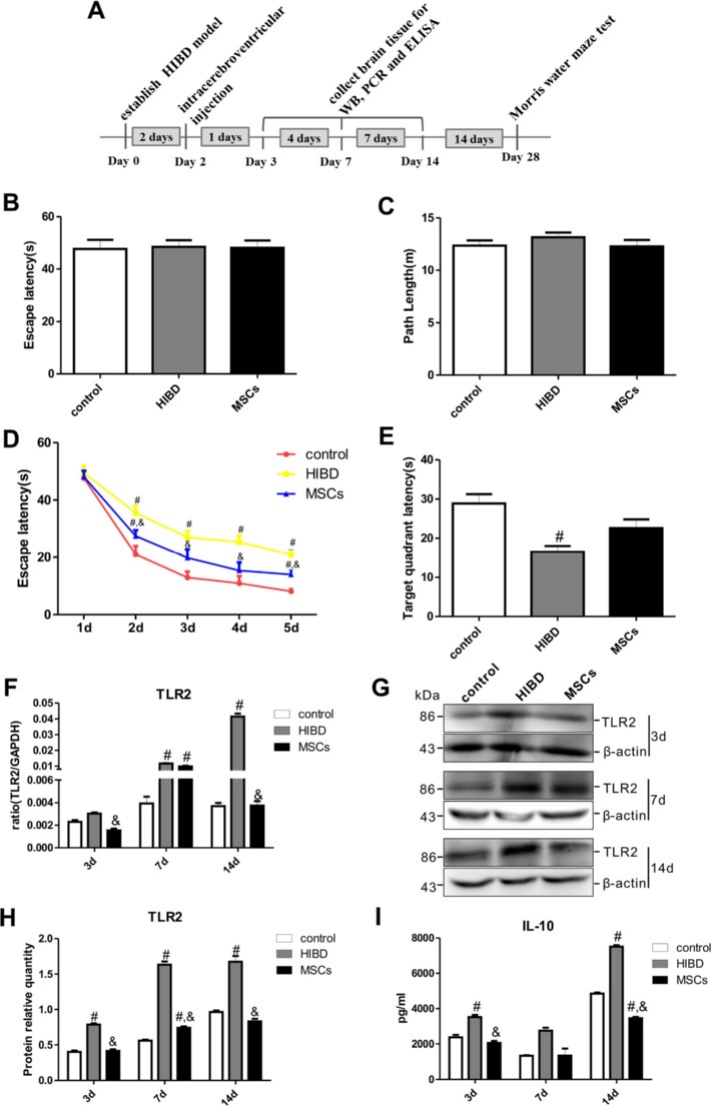
MSC transplantation improved the learning-memory function and reduced the TLR2/IL-10 expression levels of HIBD rats. **a** Diagram illustrating the experimental protocols of the treatments and tests used in the rats. **b**-**c** The escape latencies and path lengths to reach the visible platform on the first day of the Morris water maze test for the control, HIBD and MSCs rats. **d** From the 2^nd^ to the 5^th^ day of the Morris water maze test, the escape latencies to locate the visible platform gradually decreased in all three groups. **e** The duration spent in the former platform quadrant by each of the three groups on the final day of the Morris water maze test. n = 20 in each group. **f**-**g** The TLR2 mRNA and protein expression levels in the rat brains on the 3^rd^, 7^th^ and 14^th^ days following HIBD. n = 5 in each group. **h** The quantifications of WB signal in **g**. **i** The changes in the IL-10 secretion levels in the brains at the three time points after HIBD in all three groups. n = 6 in each group. ^#^ *P* < 0.05 vs. the control group; &*P* < 0.05 vs. the HIBD group

In the Morris water maze test, the escape latency and the path length required to locate the platform were recorded to assess the learning-memory function of the rats. As shown in Fig. 1b-c, all three groups exhibited similar escape latencies and path lengths during the visual training on the first day (1 d), suggesting that neither HIBD nor MSC transplantation impaired rat motility or vision. In the directional navigation experiment, the escape latencies of all groups were gradually reduced from 2 d to 5 d (Fig. 1d). However, the HIBD rats- exhibited longer escape latencies than the control rats, and the escape latency of the MSCs group- was significantly shorter than that of the HIBD group-, although the escape latency of the MSCs group did not reach the level of the control group. On the final test day, the average time that the HIBD group remained in the formerly platform-containing quadrant was the shortest among the three groups. Compared with the HIBD group, the MSCs group displayed an increased duration in this quadrant, although this difference was not significant (Fig. 1e). These results suggested that MSC transplantation partially restores the learning-memory function of neonatal HIBD rats.

Furthermore, the TLR2 mRNA levels in the brains of the HIBD rats gradually increased, and these levels were significantly different between the HIBD and control groups at both 7 d and 14 d after injury. Interestingly, the TLR2 mRNA level was significantly decreased in the MSCs group compared with the HIBD group at 14 d after injury (Fig. 1f). Western blot analysis showed the same trends for TLR2 protein expression (Fig. 1g-h). Moreover, the levels of secreted IL-10 in the brains of the HIBD group were increased at 3 d and 14 d after HIBD (Fig. 1i) and were significantly higher than those of the control group. After MSC transplantation, the IL-10 concentration in the rat brain was significantly lower than that for HIBD alone at both 3 d and 14 d after injury. These results indicated that TLR2 and IL-10 might be involved in the post-HIBD recovery process after MSC transplantation.

### TLR2 activation decreased the learning-memory function of neonatal HIBD rats

**Fig. 2:**
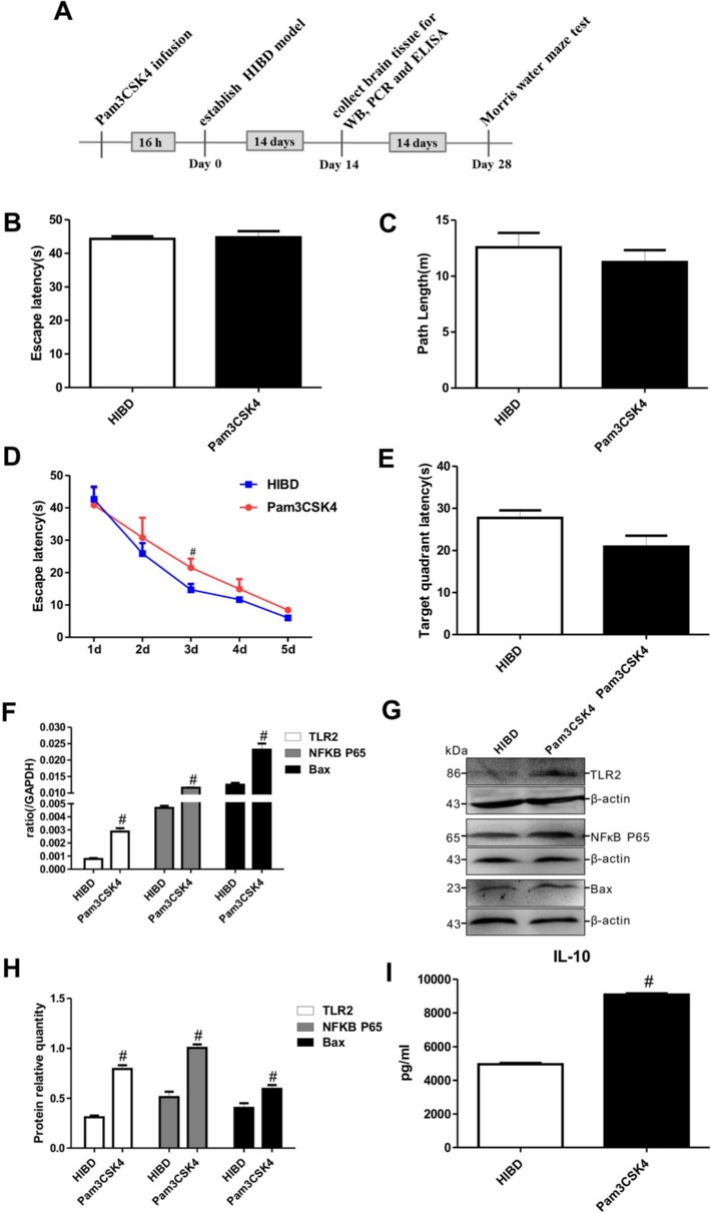
Pam3CSK4 exacerbated learning-memory dysfunction and stimulated the TLR2/NFκB signaling pathway and IL-10 secretion in HIBD rats. **a** Diagram illustrating the experimental protocols of treatments and tests used in the rats. **b**-**c** The escape latencies and path lengths to reach the visible platform on the first day of the Morris water maze test for the HIBD and Pam3CSK4 groups. **d** The escape latencies to reach the visible platform on the 2^nd^ through the 5^th^ days of the Morris water maze test for the Pam3CSK4-treated and HIBD groups. **e** The duration spent in the hidden platform quadrant by the Pam3CSK4-treated and HIBD groups. n = 20 in each group. **f**-**g** The Pam3CSK4-induced mRNA and protein expression levels of TLR2, NFκB P65 and Bax in the brains of 4-week-old rats. n = 5 in each group. **h** The quantifications of WB signal in G. **i** IL-10 release in the brains of 4-week-old rats in HIBD and Pam3CSK4 groups. n = 6 in each group. ^#^ *P* < 0.05 vs. the HIBD group

To evaluate the effects of TLR2 on the brains of HIBD rats, Pam3CSK4, a TLR2-specific agonist, was administered to neonatal HIBD rats. The results of the Morris water maze test at 1 d revealed equivalent values for both the escape latency and the path length between the HIBD and Pam3 groups (Fig. 2b-c), demonstrating that Pam3CSK4 had no effect on the motility or vision of the neonatal rats. However, the escape latency was extended in the rats that were treated with Pam3CSK4 throughout the 2 d to 5 d period of the directional navigation experiment (Fig. 2d), and the average duration spent in the former platform quadrant on the final day of the Morris water maze test tended to be lower in the Pam3CSK4 group compared with the HIBD group (Fig. 2e).

After Pam3CSK4 treatment, the levels of TLR2 mRNA and protein expression were significantly increased in the injured brain tissue, clearly indicating that Pam3CSK4 enhanced TLR2 expression *in vivo*. As shown in Fig. 2f-h, Pam3CSK4 also markedly upregulated the mRNA and protein expression of NFκB P65 and the apoptotic factor Bax compared with that for HIBD alone. Interestingly, the IL-10 concentration in the brains of the HIBD rats was significantly higher in the Pam3CSK4 group than in the HIBD group (Fig. 2i). The above results further indicated that the levels of TLR2 expression and IL-10 secretion are closely associated with the severity of injury by affecting apoptosis in HIBD rats.

### MSC transplantation reduced TLR2 expression and decreased apoptosis in the brains of HIBD rats

**Fig. 3:**
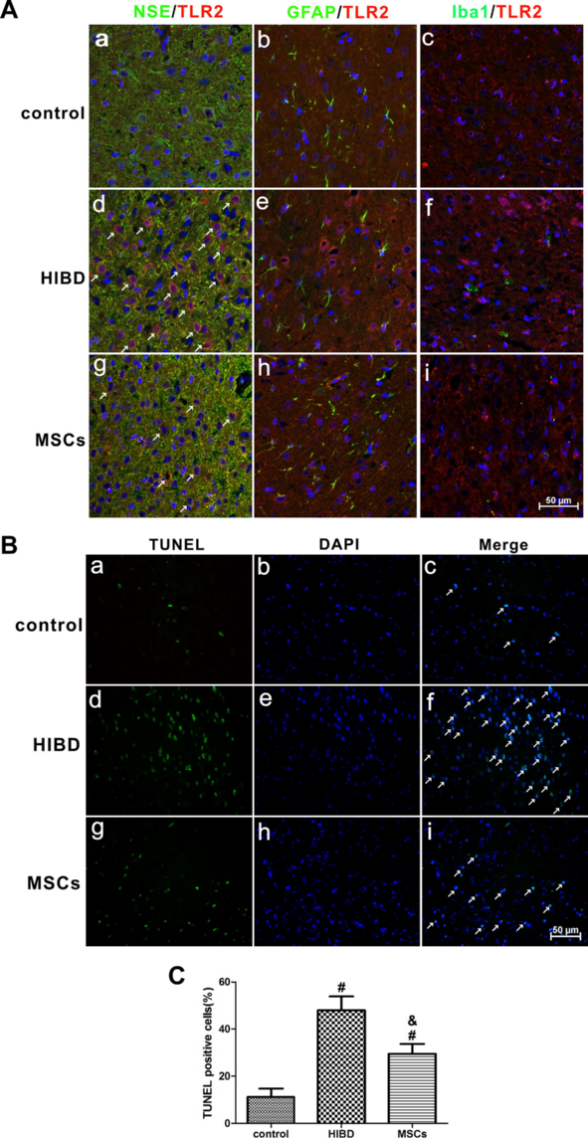
MSC transplantation decreased the expression of TLR2 in neurons and suppressed apoptosis in the cerebral cortex of HIBD rats. **a** Double immunofluorescence staining of NSE or GFAP or Iba1 (green) together with TLR2 nred) in the cerebral cortex of rats of the control, HIBD and MSCs groups. a, d and g. Double immunofluorescence staining of NSE together with TLR2. The white arrows indicate co-localization of NSE and TLR2. b, e and h. Double immunofluorescence staining of GFAP together with TLR2. c, f and i. Double immunofluorescence staining of Iba1 together with TLR2. Scale bar = 50 μm. n = 4 in each group. **b** TUNEL staining in the rat cerebral cortex of the control, HIBD and MSCs groups. a, d and g. TUNEL-positive cells. b, e and h. DAPI-stained cells. c, f and i. Merged images of TUNEL-positive cells and DAPI-stained cells. The white arrows indicate TUNEL-positive cells that were merged with DAPI-stained cells. Scale bar = 50 μm. **c** The percentage of TUNEL-positive cells in the three groups of control, HIBD and MSCs. n = 5 in each group. The results are presented as the mean ± SEM. ^#^ *P* < 0.05 vs. the control group; &*P* < 0.05 vs. the HIBD group

To evaluate the localization of TLR2 expression in the brains of HIBD rats, we used double-staining immunofluorescence to examine the cerebral cortex of three groups of rats: control, HIBD and MSCs. As shown in Fig. 3a, TLR2 was co-expressed with NSE (Fig. 3a-a, a-d and a-g) but not with GFAP (Fig. 3a-b, a-e and a-h) or Iba1 (Fig. 3a-c, a-f and a-i), suggesting that TLR2 is mainly expressed in the neuron. Meanwhile, we also observed higher TLR2 expression after HIBD (Fig. 3a-d, a-e and a-f), and MSC transplantation dramatically reduced the expression level of TLR2 (Fig. 3a-g, a-h and a-i); this result was consistent with the changes in TLR2 protein expression that were observed by Western blotting.

To further verify the effects of MSC transplantation on anti-apoptosis, TUNEL staining was performed in the brains of HIBD rats. As shown in Fig. 3b and c, the amount of TUNEL-positive cells was significantly greater in the HIBD group than in the control group, while the number of apoptotic cells was dramatically decreased following MSC transplantation-, suggesting that MSC transplantation suppressed apoptosis following HIBD.

### Co-culturing OGD-injured PC12 cells with MSCs decreased TLR2 expression and suppressed apoptosis

**Fig. 4:**
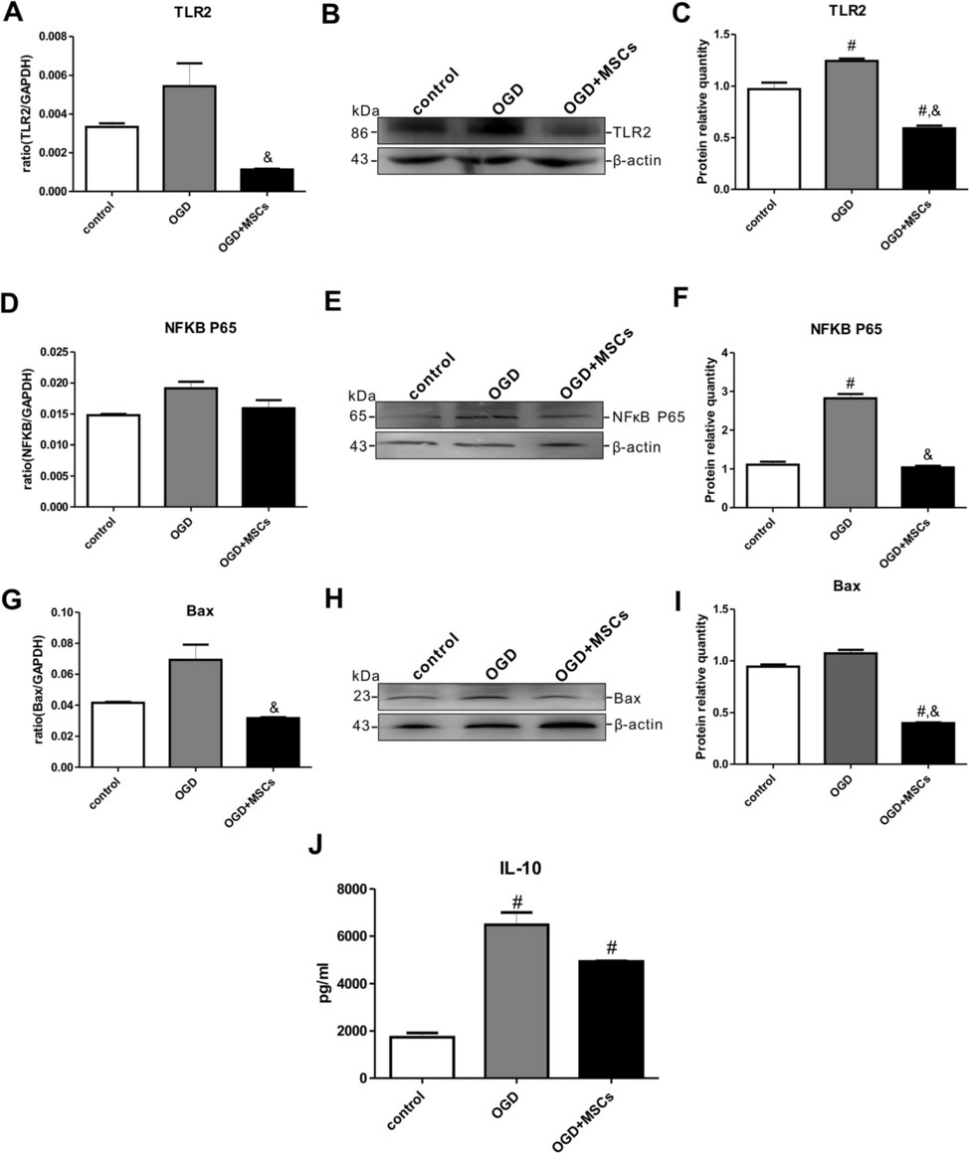
Co-culturing OGD-injured PC12 cells with MSCs decreased the expression of TLR2, NFκB, Bax and IL-10. **a**-**b** The mRNA and protein expression levels of TLR2 in OGD-injured PC12 cells in the presence or absence of MSCs. n = 6 in each group. **c** The quantifications of WB signal in **b**. **d**-**e** The mRNA and protein expression levels of NFκB P65 in OGD-injured PC12 cells in the presence or absence of MSCs. n = 6 in each group. **f** The quantifications of WB signal in **e**. **g**-**h** The mRNA and protein expression levels of Bax in OGD-injured PC12 cells in the presence or absence of MSCs. n = 6 in each group. **i** The quantifications of WB signal in **h**. **j** The concentrations of IL-10 released into the culture media were measured in the control, OGD and OGD + MSCs groups via ELISA. n = 6 in each group. ^#^ *P* < 0.05 vs. the control PC12 cells; &*P* < 0.05 vs. the OGD-injured PC12 cells

**Fig. 5:**
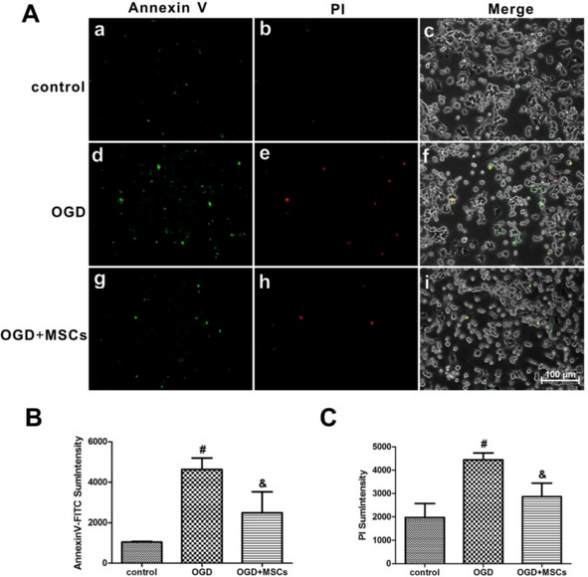
MSC co-culture suppressed apoptosis in OGD-injured PC12 cells. **a** Annexin V-FITC/PI double staining of PC12 cells in the control, OGD and OGD + MSCs groups. *a, d* and *g*. Annexin-V-positive PC12 cells in the control, OGD and OGD + MSCs groups. *b, e* and *h*. PI-positive PC12 cells in the control, OGD and OGD + MSCs groups. *c, f* and *i*. Annexin-V-positive cells and PI-positive cells merged with bright-field images of PC12 cells in the control, OGD and OGD + MSCs groups. Scale bar = 100 μm. **b**-**c** Annexin-V and PI sum intensity of PC12 cells in the control, OGD and OGD + MSCs groups. n = 5 in each group. The results are presented as the mean ± SEM. ^#^ *P* < 0.05 vs. control PC12 cells; &*P* < 0.05 vs. OGD-injured PC12 cells

To further elucidate the effects of TLR2 and IL-10 on neonatal HIBD rats, an *in vitro* OGD model was applied to PC12 cells to simulate HIBD. As shown in Fig. 4a-c, in OGD-injured PC12 cells, the TLR2 mRNA and protein expression levels were increased, and these levels were significantly decreased when the PC12 cells were co-cultured with MSCs. These findings were very consistent with the changes observed in the rat brains 14 d after HIBD. Although there was no significant difference in the NFκB P65 mRNA expression level among the control, OGD and OGD + MSCs groups (Fig. 4d), the NFκB P65 protein expression level was clearly increased in the OGD group and reduced in the OGD + MSCs group (Fig. 4e-f). Both the mRNA and protein expression levels of Bax were also significantly increased after OGD treatment and were significantly decreased by MSC co-culture (Fig. 4g-i). In addition, as shown in Fig. 5a, the numbers of both annexin V-positive and PI-positive cells were significantly higher in PC12 cells injured by OGD compared with those of the uninjured group (Fig. 5a a-f, b-c). These values were obviously decreased in OGD PC12 cells after MSC co-culture (Fig. 5a g-i, b-c). These results indicated that the separate co-culture with MSCs protected OGD-injured PC12 cells from apoptosis via the TLR2/NFκB pathway. Furthermore, the level of IL-10 secreted from PC12 cells following OGD exposure was significantly increased by more than 3-fold compared with that of non-OGD-exposed cells. In OGD-injured PC12 cells co-cultured with MSCs, the release of IL-10 was clearly alleviated compared with that in non-cultured OGD-injured PC12 cells, but a significant difference between the control and OGD + MSCs groups remained (Fig. 4j). These results further demonstrated that IL-10 might be involved in the regulation of TLR2 following neuronal damage.

### TLR2 pathway in PC12 cells after Pam3CSK4 or Ad-siTLR2 treatment

**Fig. 6:**
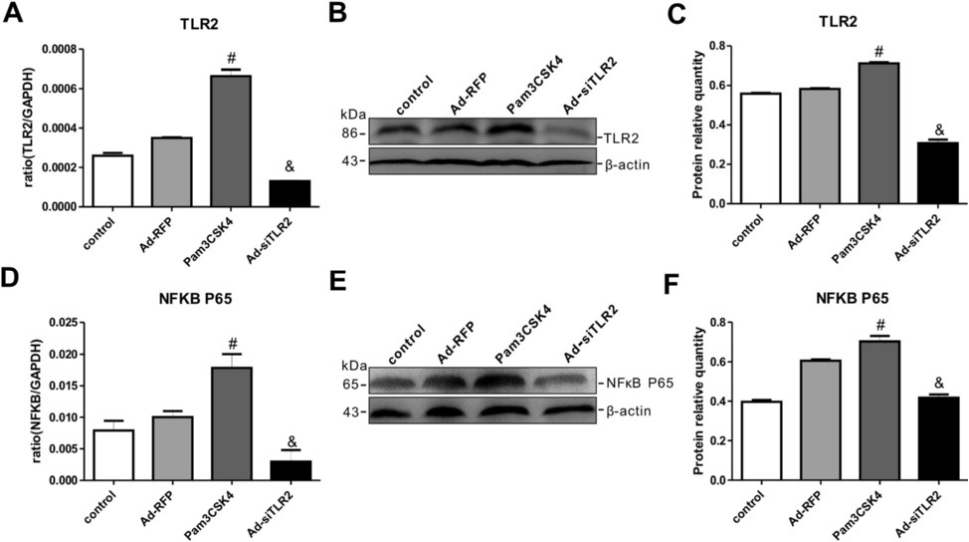
Pam3CSK4 effectively activated and Ad-siTLR2 specifically blocked the TLR2/NFκB signaling pathway in PC12 cells. **a**-**b** The mRNA and protein expression levels of TLR2 in PC12 cells following Pam3CSK4 treatment or Ad-siTLR2 infection. **c** The quantifications of WB signal in **b**. n = 6 in each group. **d**-**e** The mRNA and protein expression levels of NFκB in PC12 cells following Pam3CSK4 treatment or Ad-siTLR2 infection. **f** The quantifications of WB signal in **e**. n = 6 in each group. ^#^ *P* < 0.05 vs. untreated PC12 cells; &*P* < 0.05 vs. Ad-RFP-infected PC12 cells

To confirm the regulatory role of TLR2 in neuronal injury, Pam3CSK4 or siTLR2 was applied to PC12 cells. As shown in Fig. 6a-c, Pam3CSK4 significantly upregulated TLR2 mRNA and protein expression in PC12 cells compared with that in non-Pam3CSK4–treated control cells-, whereas Ad-siTLR2 treatment significantly downregulated the TLR2 expression levels in PC12 cells compared with those of Ad-RFP treatment group. Additionally, we measured the expression levels of NFκB in PC12 cells after Pam3CSK4 or Ad-siTLR2 treatment. As shown in Fig. 6d-f, the changes in the mRNA and protein expression levels of NFκB completely mirrored those of TLR2 following Pam3CSK4 or Ad-siTLR2 treatment. The above results revealed that Pam3CSK4 or Ad-siTLR2 treatment not only specifically activated and inhibited TLR2 expression, respectively, but also effectively induced and impaired the TLR2 signaling pathway, respectively, in PC12 cells.

### MSCs protected against TLR2 signaling pathway-mediated apoptosis in PC12 cells subjected to OGD injury

**Fig. 7:**
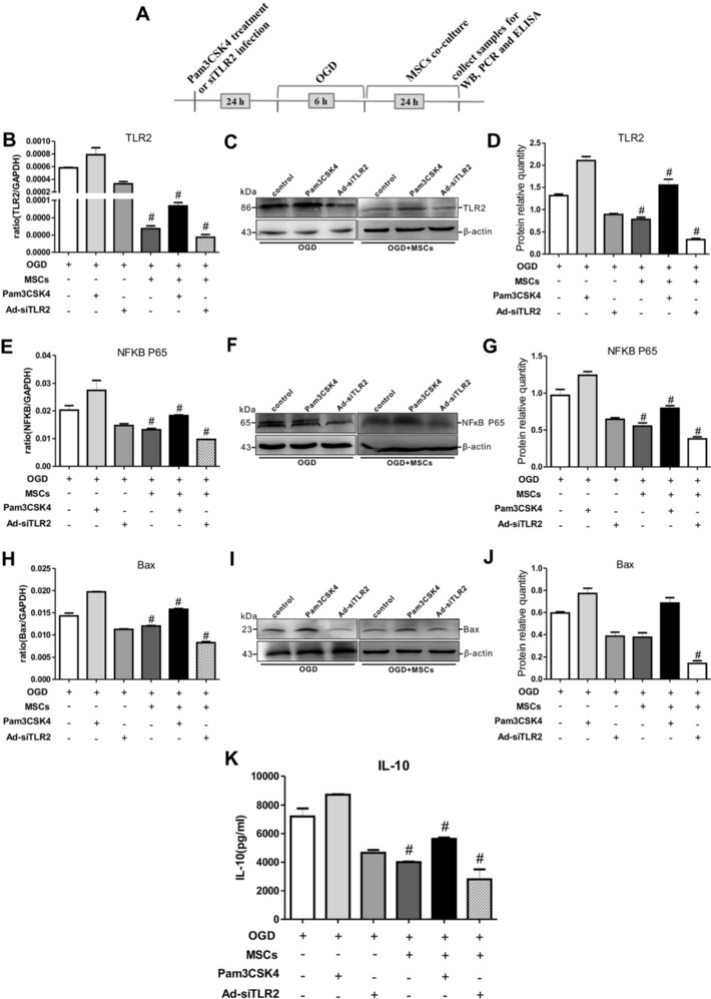
MSCs modulated the TLR2/NFκB signaling pathway and suppressed the apoptosis of OGD-injured PC12 cells and the secretion of IL-10. **a** Diagram illustrating the experimental protocols of the treatments and tests for PC12 cells. **b**-**c** The TLR2 mRNA and protein expression levels in co-cultures of MSCs with OGD-injured PC12 cells that received Pam3CSK4 or Ad-siTLR2 treatment. n = 6 in each group. **d** The quantifications of WB signal in **c**. **e**-**f** The mRNA and protein expression levels of NFκB P65 in co-cultures of MSCs with OGD-injured PC12 cells that were treated with Pam3CSK4 or Ad-siTLR2. n = 6 in each group. **g** The quantifications of WB signal in **f**. **h**-**i** The changes in the mRNA and protein expression levels of Bax in co-cultures of MSCs with OGD-injured PC12 cells that were treated with Pam3CSK4 or Ad-siTLR2. n = 6 in each group. **j** The quantifications of WB signal in **i**. **k** The changes in IL-10 release in co-cultures of MSCs with OGD-injured PC12 cells that received Pam3CSK4 or Ad-siTLR2 treatment. n = 6 in each group. ^#^ *P* < 0.05 vs. OGD-injured PC12 cells that were treated with Pam3CSK4 or Ad-siTLR2

To investigate whether the TLR2 pathway is critically involved in the enhanced recovery from OGD exposure that is mediated by MSCs, Pam3CSK4 or Ad-siTLR2 treatment was applied to the *in vitro* co-culture model of MSCs and OGD-injured PC12 cells. As shown in Fig. 7b-d, TLR2 mRNA and protein expression levels were increased by Pam3CSK4 treatment and reduced by Ad-siTLR2 treatment in OGD-injured PC12 cells in the presence or absence of MSCs. However, compared with the situation for OGD injury in the absence of MSCs, the TLR2 expression levels in PC12 cells were significantly decreased following OGD in the presence of MSCs when treated with either Pam3CSK4 or Ad-siTLR2-. Moreover, the changes in NFκB expression were similar to those of TLR2 in all groups regardless of the presence of MSCs (Fig. 7e-g). These results suggested that co-culturing the PC12 cells with MSCs could modulate the TLR2 signaling pathway via NFκB, thereby alleviating OGD-mediated damage to PC12 cells.

Intriguingly, consistent with the increase in TLR2 expression, the Bax mRNA and protein expression levels were increased by the application of Pam3CSK4 to OGD-injured PC12 cells. In contrast, the Bax expression levels were reduced by Ad-siTLR2 treatment (Fig. 7h-j). Co-culturing PC12 cells with MSCs dramatically suppressed the Bax expression levels in OGD-injured PC12 cells compared with those in monocultured OGD injured-PC12 cells in the presence of either Pam3CSK4 or Ad-siTLR2-. These findings revealed that co-culturing these cells with MSCs might reduce apoptosis in injured cells via TLR2.

Surprisingly, we found that the changes in the IL-10 concentrations were highly consistent with the changes in the TLR2 and Bax expression levels. As shown in Fig. 7k, Pam3CSK4 treatment enhanced the IL-10 release from OGD-injured PC12 cells, whereas Ad-siTLR2 infection reduced the IL-10 release. However, in MSC co-cultures, the IL-10 concentrations were significantly suppressed in OGD-injured PC12 cells that were treated with either Pam3CSK4 or Ad-siTLR2-. These findings indicated that TLR2 might directly or indirectly regulate the secretion of IL-10 into the microenvironment surrounding injured PC12 cells and that co-culturing these cells with MSCs decreases the release of IL-10 by modulating the TLR2 pathway.

## Discussion

In the present study, we sought to clarify whether MSC transplantation modulates TLR2 signaling and improves the recovery of learning-memory function following HIBD and the possible mechanism(s) underlying these effects. The primary findings of this study were that MSC treatment ameliorated the deficits in learning-memory function and decreased the TLR2 expression levels in the brain of HIBD rats via suppression of the TLR2–dependent NFκB signaling pathway to decrease apoptosis and IL-10 release.

Substantial evidence indicates that MSC treatment improves behavioral outcomes following HIBD [6–10, 20–22]. The present study also demonstrated that MSC transplantation effectively alleviated the impairment of the learning-memory function of neonatal HIBD rats. Concurrently, we found that MSCs suppressed the hypoxia-ischemia–induced TLR2 expression in the brains of HIBD rats. These results indicated that TLR2 might be involved in the recovery of learning-memory function after MSC transplantation into HIBD rats. To date, several studies have demonstrated that MSCs alleviate neuronal dysfunction via the differentiation of MSCs into neurons at the lesion site [11, 12] because only a very small percentage of MSCs differentiate into a parenchymal cell phenotype [23]. Therefore, increasing numbers of investigators have shifted their focus to the immunomodulatory functions of MSCs that stimulate the release of trophic factors in the injury microenvironment [7]. Previous studies have demonstrated that MSC treatment represses inflammation and facilitates the development and integration of new neurons by decreasing the levels of pro-inflammatory cytokines, which are secreted from activated microglia in the ischemic brain [9, 24]. Nevertheless, none of these studies has demonstrated the effects of MSC transplantation on TLR2 signaling in the HIBD brain.

TLRs are a family of innate immune system receptors that respond to diverse pathogen-derived and injury-induced endogenous ligands [17]. TLR2 is upregulated and activated in neurons in response to ischemia, and focal cerebral ischemic injury is alleviated in TLR2-deficient mice [25, 26]. To confirm the effect of TLR2 on learning-memory function, neonatal rats were injected with Pam3CSK4 prior to HIBD. The results indicated that the activation of the TLR2/NFκB signaling pathway exacerbates the effects of HIBD, including apoptosis. These findings are consistent with those of an *in vitro* study of the amyloid β peptide-induced inflammatory response in mouse NG108-15 neural cells [27]. Some studies have found that heat shock proteins (HSPs) in damaged tissue activate the TLR2-dependent NFκB signaling pathway to promote the expression of the cytokines tumor necrosis factor-α (TNFα), IL-1β and IL-6 [28], which participate in inflammatory injury to induce neuronal apoptosis. A study by Kim also demonstrated that HSP60 increases the TLR2 expression level and induces TLR-dependent apoptosis via activation of the NFκB pathway [29]. Therefore, we hypothesized that MSC transplantation might alleviate the apoptosis induced by HIBD via the suppression of the TLR2/NFκB signaling pathway.

The PC12 cell line has been widely used as an *in vitro* model to examine neurological diseases [30–32]. To confirm that MSCs suppress the TLR2 signaling pathway and inhibit apoptosis following HIBD, we utilized an *in vitro* OGD model in PC12 cells. The data demonstrated that co-culturing PC12 cells with MSCs repressed the activation of TLR2/NFκB signaling, thereby decreasing apoptosis in the OGD-injured PC12 cells; this finding indicated that MSCs might play a protective role via the immunosuppression of the TLR2/NFκB signaling pathway to reduce OGD-induced apoptosis. These results are consistent with our findings in a previous study showing that apoptosis is suppressed in MSC co-culture via the modulation of the microenvironment surrounding the injured neurons [33]. However, it should be noted that this study only demonstrated that MSC transplantation modulates the TLR2/NFκB signaling pathway during HIBD injury. Further studies should focus on the mechanism underlying the MSC-mediated immunomodulation of TLR2.

IL-10 limits the production of pro-inflammatory cytokines and chemokines [34]. More severe damage and even death can occur in acutely infected hosts if IL-10 signaling is blocked [35]. In the current study, we were surprised to find that the pattern of changes in the IL-10 secretion levels was highly consistent with the alterations in TLR2 expression both *in vivo* and *in vitro*. Our results revealed that IL-10 levels were significantly increased in both HIBD rats and OGD-injured PC12 cells and were significantly alleviated by MSC transplantation or co-culture, respectively. Moreover, Pam3CSK4 treatment enhanced IL-10 release both *in vivo* and *in vitro*, whereas Ad-siTLR2 infection suppressed IL-10 secretion. These changes in IL-10 release suggested that the MSC transplantation-mediated effect on IL-10 secretion is regulated by TLR2/NFκB signaling in HIBD rats. Yang et al. found that adult neural stem cells (aNSCs) that were engineered to express IL-10 were better able to induce immunosuppression, remyelination and neuronal repair [21]. Based on the combination of our discoveries regarding the neuroprotective effect of MSCs and the results of their study, we speculate that MSCs suppress apoptosis and alleviate HIBD injury by decreasing the levels of pro-inflammatory cytokines in the injury microenvironment, which provides feedback that reduces IL-10 secretion. Currently, we are investigating the effects of IL-10 during MSC transplantation therapy on HIBD using siRNA targeting IL-10.

## Conclusions

Our results demonstrated that TLR2 is upregulated following HIBD and that MSC transplantation enhances the recovery of learning-memory function in HIBD rats and plays a neuroprotective role by reducing the apoptosis of damaged nerve cells via the suppression of the TLR2/NFκB signaling pathway. Additionally, we revealed that the cytokine IL-10 is involved in HIBD and is regulated by the TLR2/NFκB pathway following the administration of MSCs. These findings might provide new evidence supporting the use of MSC therapies for HIBD.

## Materials and methods

### Animal groups

All animal experimental protocols were approved by the Animal Experimentation Ethical Committee of the Zoology Center at Chongqing Medical University (Chongqing, China). Specific pathogen-free (SPF)-grade Wistar rats (6 weeks old) were purchased from the Experimental Animal Centre of Daping Hospital at the Third Military Medical University (Chongqing, China), and the SPF-grade animal housing was certified for experimental animals (SYXK(Yu)2012-0015). Seven-day postnatal rats were used to establish the HIBD model as reported previously [36, 37]. Briefly, left carotid artery ligation was carried out in 7-day-postnatal rats. Two hours later, the pups were exposed to 8 % oxygen at 37 °C for 2.5 h and then they were returned to their dams.

All pups were randomly separated into one of the following four groups: control, HIBD, MSCs, and Pam3CSK4. The rats in the control group were only subjected to a cervical skin incision that was subsequently sutured. The HIBD pups in the MSCs group were transfused with 1.5 × 10^6^ MSCs in 5 μL of phosphate-buffered saline (PBS) (Hyclone, USA) via intracerebroventricular injections 2 days after hypoxia-ischemia [37]. The rat primary MSCs that were used were isolated and expanded as described previously [38]. Briefly, the primary MSCs were isolated from the bone marrow in leg bones of 3 to 4-week-old rats. The cells were cultured with DMEM/F12 supplemented with 10 % fetal bovine serum (FBS). The medium was replaced with fresh medium every 2-3 days. The pups in the Pam3CSK4 group were infused with 30 μg of Pam3CSK4 16 h before hypoxic-ischemic injury.

### Morris water maze test

Four weeks after the treatments, 15 rats per group were subjected to the Morris water maze task (MWM SLY-WMS 2.0, China) to evaluate their spatial learning and memory functions as reported previously [39].

### The OGD cell model and the co-culture systems

PC12 cells were purchased from the Cell Bank of the Shanghai Institute of Cell Biology at the Chinese Academy of Sciences (Shanghai, China). The cells were cultured as described previously [33]. At approximately 90 % confluence, the PC12 cell culture medium was replaced with Earle’s balanced salt solution (EBSS) (Hyclone, USA). Then, the cells were placed in an incubator (Thermo Forma 3111, Thermo Scientific) containing 5 % O2 and 95 % N2 at 37 °C for 6 h. Normal PC12 cells that were not subjected to OGD served as controls. A total of 2 × 10^6^ MSCs were added to Dulbecco’s modified Eagle’s medium (DMEM) (Hyclone, USA) to generate co-cultures with the OGD-injured PC12 cells, and the cells treated in this manner were referred to as the OGD + MSCs group.

### PC12 cell treatments

For Pam3CSK4 treatment, normal PC12 cells were treated with 300 ng/ml Pam3CSK4 (InvivoGen at the time of OGD injury, and cells treated with an equal volume of PBS served as the controls. For the Ad-siTLR2 group, normal PC12 cells at 80 % confluence were infected with siTLR2 adenovirus [40] for 24-48 h, and PC12 cells subjected to an equivalent infection load of RFP-expressing adenovirus served as the controls.

For the three OGD groups that were subjected to different treatments, the PC12 cells were subjected to OGD for 6 h after Pam3CSK4 treatment or after siTLR2 infection for 24 h. Untreated PC12 cells that were subjected to OGD were used as negative controls. The PC12 cells that were treated with Pam3CSK4 or siTLR2 were co-cultured with MSCs for an additional 24 h following OGD. Untreated cells were exposed to OGD and co-cultured with MSCs for use as negative controls for the other MSC co-culture groups.

### RNA extraction and real-time PCR

The sample preparation and PCR reaction protocols were identical to those of a previous report [33]. The rat TLR2, NFкB P65, Bax and glyceraldehyde 3-phosphate dehydrogenase (GAPDH) primer sequences were designed using Primer 5 software and are listed in Table 1. The Ct values derived from the real-time PCR data were utilized to ensure that an individual PCR product was amplified in each reaction. All samples were normalized to the endogenous levels of GAPDH.

**Table 1:** Primers of the target genes used for real-time PCR

Primers	Sequences
TLR2	F: TCTTGATGGCTGTGATAGG
	R: CCGAGGGAATAGAGGTGA
NFκB P65	F: AGGACTGCCGGGATGGCTTCTAT
	R: GGTCTGGATGCGCTGGCTAATGG
Bax	F: AAGTAGAAGAGGGCAACCAC
	R: GATGGCAACTTCAACTGGG
GAPDH	F: CCTGGAGAAACCTGCCAAG
	R: CACAGGAGACAACCTGGTCC

### Western blot

Total protein was extracted using a specific extraction kit (Bioteke Co., Ltd.). Approximately 50 μg of total protein per well was loaded on a 10 % sodium dodecyl sulfate (SDS)-polyacrylamide gel (Beyotime). The protein isolation procedure corresponded to the conventional method used for Western blot. The anti-TLR2 (1:1000, Abcam, ab13855), anti-NFкB P65 (1:500, Santa Cruz, sc-8008), anti-Bax (1:500, Santa Cruz, sc-7480) and anti-β-actin primary antibodies (1:500, Santa Cruz, sc-130065) were incubated at 4 °C overnight. The next day, the membranes were probed with horseradish peroxidase (HRP)-conjugated secondary antibodies (1:5000, Santa Cruz) at room temperature for 1 h, and the chemiluminescence of the protein bands was measured using a Syngene GBox Imaging System.

### Enzyme-linked immunosorbent assay (ELISA)

The levels of IL-10 cytokine release in the brain tissues and the culture media from the various treatment groups were measured using an ELISA kit (Raybiotech, Inc., Norcross, GA, USA). The colorimetric absorbance was recorded at a wavelength of 450 nm. The IL-10 concentrations were calculated according to a standard curve constructed for each assay, and each assay was performed in triplicate.

### TUNEL assay and immunofluorescence staining

TUNEL assay and immunofluorescence double-labeling staining of TLR2 with NSE, GFAP or Iba1 were performed on the brains of rats in the control, HIBD and MSCs groups. Briefly, all rats were anesthetized and transcardially perfused with 4 % paraformaldehyde (Kelong Chemical Co. Ltd, China). The 12-μm slices of brain tissue were sectioned using a Leica CM3050 S cryostat. Apoptosis was detected using a TUNEL kit (KeyGEN BioTECH, China) following the manufacturer’s protocol. The number of TUNEL-positive cells was counted from four randomly selected fields in each group, and the counts were expressed as a percentage of the total number of cells.

Double stainings for TLR2 and NSE, GFAP or Iba1 were performed to evaluate their colocalization by using the following antibodies: goat anti-TLR2 (1:50, Santa Cruz, sc-16237) together with rabbit anti-NSE (1:100, Abcam, ab53025) or mouse anti-GFAP (1:100, Abcam, ab10062) or rabbit anti-Iba1 (1:50, Santa Cruz, sc-32725) overnight at 4 °C. The primary antibodies were visualized using Alexa Fluor 488-conjugated chicken anti-rabbit (anti-mouse) IgG (1:100, Life Technologies) together with Alexa Fluor 594-conjugated donkey anti-goat IgG (1:100, Jackson), and 4’,6-diamidino-2-phenylindole (DAPI, 1:100, Yeasen) was used to stain the nucleus. The images were captured using a Nikon laser confocal microscope (Nikon, Japan).

### Apoptosis assay

Apoptosis of PC12 cells in the control, OGD and OGD + MSCs groups was detected using an annexin V-FITC/PI double-staining kit (KeyGen BioTECH) according to the manufacturer’s recommendations. Briefly, after treatment, the cells in the three groups were washed twice with PBS buffer and then incubated with 5 μl of FITC-conjugated annexin V and 5 μl of PI in 500 μl of binding buffer for 5 min at room temperature. Apoptotic changes in PC12 cells with different treatments were observed under an inverted fluorescence microscope (Nikon, Japan) and photographed using a digital camera.

### Statistical analyses

The data are expressed as the means ± SEM. Significant differences between the groups were determined via one-way repeated-measures analyses of variance (ANOVAs) followed by Duncan’s multiple range tests. All statistical analyses were performed using GraphPad Prism 5.0 software (GraphPad Software, Inc., USA). *P*<0.05 was considered to be significant.

## Abbreviations

DMEM: Dulbecco’s modified eagle medium
EBSS: Earle’s balanced salt solution
ELISA: Enzyme-linked immunosorbent assay
GAPDH: Glyceraldehyde 3-phosphate dehydrogenase
HIBD: Hypoxic-ischemic brain damage
HRP: Horse reddish peroxidase
HSP: Heat shock proteins
IL-1β: Inerlukin-1β
IL-6: Inerlukin-6
IL-10: Interlukin-10
MSCs: Mesenchymal stem cells
NFκB: Nuclear factor kappa B
OGD: Oxygen glucose deprivation
PBS: Phosphate-buffer saline
PC12: Adrenal pheochromocytoma
SDS: Sodium-dodecyl sulphate
SPF: Specified pathogen-free
TLR2: Toll-like receptor 2
TNF-α: Tumor necrosis factor-α
